# Optimizing Adherence to Oral Anticancer Agents: Results from an Implementation Mapping Study

**DOI:** 10.3390/curroncol32020078

**Published:** 2025-01-29

**Authors:** Benyam Muluneh, Maurlia Upchurch, Emily Mackler, Ashley Leak Bryant, William A. Wood, Stephanie B. Wheeler, Leah L. Zullig, Jennifer Elston Lafata

**Affiliations:** 1 Division of Pharmacotherapy and Experimental Therapeutics, Eshelman School of Pharmacy, University of North Carolina at Chapel Hill, Chapel Hill, NC 27599, USA; 2 Lineberger Comprehensive Cancer Center, University of North Carolina at Chapel Hill, Chapel Hill, NC 27599, USA; 3 Michigan Oncology Quality Consortium, Ann Arbor, MI 48105, USA; 4 School of Nursing, University of North Carolina at Chapel Hill, Chapel Hill, NC 27599, USA; 5 School of Medicine, University of North Carolina at Chapel Hill, Chapel Hill, NC 27599, USA; 6 Department of Health Policy and Management, Gillings School of Global Public Health, University of North Carolina at Chapel Hill, Chapel Hill, NC 27599, USA; 7 Department of Population Health Sciences, Duke University School of Medicine, Durham, NC 27710, USA; 8 Center of Innovation to Accelerate Discovery and Practice Transformation, Durham Veterans Affairs Health Care System, Durham, NC 27705, USA; 9 Division of Pharmaceutical Outcomes and Policy, Eshelman School of Pharmacy, University of North Carolina at Chapel Hill, Chapel Hill, NC 27599, USA

**Keywords:** oral chemotherapy, oral anticancer agents, medication adherence, implementation mapping

## Abstract

Clinical trials inform cancer care, yet real-world outcomes often diverge due to patient-related factors, like age, organ dysfunction, and nonadherence to oral anticancer agents (OAAs). While oncology organizations emphasize patient support programs, practical guidance on designing and implementing these programs is limited. We conducted a two-phase, mixed-methods study to enhance the adoption, implementation, and sustainability of an OAA adherence program (OAP). In phase 1, we used implementation mapping (IM) with a multidisciplinary expert panel to develop six strategies: (1) memorandum of understanding (MOU), (2) data-driven presentation, (3) standard operating procedures (SOPs), (4) motivational interviewing (MI) training, (5) electronic health record (EHR) templates, and (6) key performance indicators (KPIs). In phase 2, oncology professionals (n = 34) completed surveys, and a subset (n = 10) participated in interviews to assess feasibility, acceptability, and appropriateness. EHR templates and SOPs were rated as the most feasible and acceptable strategies, while MI training and formal agreements received moderate ratings. Interviews highlighted the importance of leadership buy-in, incremental implementation, and clear documentation. Participants valued KPIs for tracking adherence and outcomes but noted resource constraints and staff workload as challenges. Using IM, we co-developed strategies to activate OAA adherence-focused clinical programs. Tools standardizing care, like EHR templates and SOPs, were highly endorsed. Future work will test these strategies in a hybrid trial to improve real-world oncology outcomes.

## 1. Introduction

Clinical trials often result in the discovery and refinement of new therapies that can improve the efficacy and safety of cancer care. However, these findings are often not replicated in practice [1,2]. The existing data show that safety outcomes are compromised partly because patients in clinical practice (i.e., “real-world” settings) tend to be older and have higher rates of organ dysfunction, leading to reduced drug clearance and subsequent adverse reactions [3,4]. Medication nonadherence may also threaten real-world effectiveness, which may occur as a result of multiple overlapping factors, including patient-related factors (e.g., forgetfulness and a lack of knowledge of benefits) and system-related factors (e.g., pharmacy shipment delays stemming from insurance pre-authorization requirements, supply chain challenges, etc.) [5,6,7,8]. Professional oncology organizations [9,10,11] have indicated the importance of instituting patient support programs to improve adherence and other patient-centered outcomes; however, how best to design and implement such programs remains understudied.

Our team designed and successfully piloted an interdisciplinary OAA adherence-support program (OAP) that included pre-treatment patient education by pharmacists, medication access services by designated technicians, and ongoing side-effect monitoring by clinicians (including clinical pharmacists and other advanced practice providers). The program improved medication adherence rates and was received positively by the patients and providers [12]. However, the program—which has not been replicated elsewhere—was not sustained beyond the pilot phase. The results from a formative evaluation identified key barriers that hampered the program’s adoption, implementation, and sustainment [13]. The six key barriers included: (1) physicians’ lack of awareness of details about prior adherence programs; (2) administrators’ concerns about program costs; (3) confusion regarding roles/responsibilities; (4) low staff self-efficacy helping patients to adhere to medications; (5) lack of discrete measures to track patient adherence and other patient reported outcomes; and (6) no clear measures to define program success [13]. To address these barriers, our team used a modified implementation mapping (IM) approach—a five-step planning framework—to produce concrete implementation strategies that would facilitate the adoption, implementation, and sustainment of an OAP [14]. Here, we describe the process for IM-guided strategy development (phase 1), as well as the results of our mixed-methods study on clinicians’ perceptions of the feasibility, acceptability, and appropriateness of utilizing the designed strategies (phase 2).

## 2. Materials and Methods

**Figure 1:**
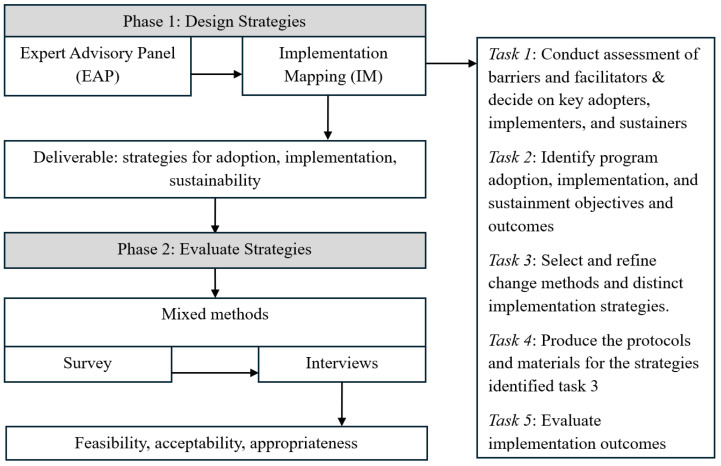
Study schema. In phase 1 of the study (design strategies), we assembled a diverse team of clinicians, administrators, and staff as part of our expert advisory panel (EAP). This EAP, with the guidance of the project team, worked through the tasks of implementation mapping (IM) in order to generate concrete strategies to support the adoption, implementation, and sustainment of our adherence intervention [12]. In phase 2 of the study (evaluation of strategies), we conducted a mixed-methods study where we surveyed and interviewed oncology clinicians and hospital administrators. The primary goal of phase 2 was to elicit participants’ perceptions of the designed strategies with regards to feasibility, acceptability, and appropriateness [15].

We conducted a two-phase, sequential explanatory mixed-methods study to design and evaluate concrete strategies to facilitate: (1) the adoption, (2) implementation, and (3) sustainment of an OAP for patients with cancer (Figure 1). In phase 1, we designed concrete strategies to overcome pre-identified adoption and implementation barriers [13]. Subsequently, in phase 2, we conducted a mixed-methods study (survey and interview) to evaluate the feasibility, acceptability, and appropriateness of the strategies developed in phase 1 to guide further strategy refinement prior to a future pilot.

This study was determined to be exempt from UNC’s Institutional Review Board (IRB).

### 2.1. Phase 1. Implementation Mapping (IM): Design Concrete Strategies to Enhance OAP Adoption, Implementation, and Sustainment

In phase 1, we assembled a multidisciplinary expert advisory panel with representation from three entities affiliated with UNC Health, an integrated health system in North Carolina that includes a centrally located academic medical center and eight affiliated community facilities with dedicated oncology programs. The expert advisory panel included two hematologists, one nurse practitioner, two clinical pharmacists, four administrators, three information and technology (IT) professionals, and one social worker from the academic facility; one hematologist, two pharmacists, and one administrator from a community facility; and two managers from the system’s affiliated specialty pharmacy. Using a modified nominal group technique—a multistep method to generate and prioritize ideas through brainstorming, discussion, and voting/ranking—we executed five tasks of implementation mapping (IM) with the expert advisory panel. For task 1, we reviewed the list of barriers identified through semi-structured interviews in a prior study [13]. We also identified a list of key personnel to determine who needed to do what (e.g., adopters, such as the cancer center director and pharmacy director, would need to decide to adopt the OAP; implementers, such as physicians, pharmacists, and nurses, would need to execute the OAP; and sustainers, such as the cancer center quality improvement committee, would need to maintain the OAP). In task 2, we defined the program outcomes and objectives. In tasks 3 and 4, we selected and produced distinct implementation strategies to enhance the adoption, implementation, and sustainment of the OAP. In task 5, we defined a program evaluation plan. Throughout the IM process, we used nominal group technique principles to brainstorm and record ideas with the expert advisory panel (four 60 min virtual meetings), discuss and refine ideas to build a consensus (three 60 min virtual meetings), and vote on final ideas via an electronic survey disseminated via email (e.g., to decide on program objectives, key strategies, etc.). Meetings were facilitated by the study’s principal investigator (BM), a board-certified clinical pharmacist with experience designing pharmaceutical care programs.

### 2.2. Phase 2. Mixed-Methods Study: Evaluate the Feasibility, Acceptability, and Appropriateness of Strategies from Phase 1

In phase 2, we used a sequential explanatory mixed-method approach: first we designed and disseminated a survey for oncology clinicians within UNC Health to gather feedback on the feasibility, appropriateness, and acceptability of the recommended distinct strategies developed in phase 1 (QUANT); next, we conducted individual semi-structured interviews from a volunteer subgroup of the surveyed individuals to elicit additional insights and clarifications regarding the feasibility of adopting and implementing the strategies identified in phase 1 (QUAL).

Survey: Clinicians and hospital administrators were recruited via email from academic, rural, and urban community cancer settings in North Carolina to complete a survey via Qualtrics^®^ (Seattle, WA, USA). Eligible participants were identified from a systematic search of oncology practice sites in North Carolina. The survey consisted of an introduction to the study, demographic questions, and detailed descriptions of each strategy followed by questions on feasibility, acceptability, and appropriateness of implementation. The survey was adapted from Weiner’s three outcome measures: Feasibility of Intervention Measure (FIM), Acceptability of Intervention Measure (AIM), and Intervention Appropriateness Measure (IAM) [15]. Subdomains for each of the measures included an assessment of whether the strategy was perceived to be possible, implementable, doable, and easy to use for the FIM; a good match, fitting, applicable, and suitable for the AIM; and appealing, likeable, and welcoming for the IAM. In addition to these measures, we asked how strongly respondents agreed with statements regarding the inclusion of specific components of each strategy. For each item, we used 5-point Likert scale responses with open-ended fields for respondents to note additional comments and specific recommendations for improvement. We used descriptive statistics to summarize the data.

Interviews: Survey participants were then recruited via a question asking if they would be interested in also completing qualitative interviews. Among those volunteering, we used a stratified random sampling process to ensure representation from clinicians, administrators, and practice settings (academic vs. community). Using purposive sampling, individuals were invited by e-mail to participate in one-on-one interviews lasting approximately 45 min each. Those completing interviews were provided with USD 50 gift cards as a token of appreciation for their time. All interviews were conducted via Zoom with videoconferencing. Participants were introduced to the study, asked about their work and organization, and then asked to react to examples of half of the phase 1 strategies in alternating groups to optimize efficiency, allow for selection variation, and prevent participant fatigue. Key evaluation queries included: whether this strategy is feasible for clinicians to use, if there is any information that may be required to get buy-in from leadership, and what challenges clinicians may face in implementing this strategy at their organization. Interviews were conducted by a trained research assistant at UNC’s Odum Institute for Research in Social Science.

The interviews were recorded and transcribed with the participants’ permission. Th e data gathered through the interviews were organized via MAXQDA (v.2022), a qualitative data analysis software. A codebook was developed with a priori and emergent codes. Quotes for each code were examined, and matrices and memos were used to organize and examine the data for themes and analyses. We employed a data integration table to synthesize the findings by aligning qualitative quotes directly with corresponding quantitative results, facilitating a comprehensive understanding of the data.

## 3. Results

### 3.1. Phase 1: Implementation Mapping

#### 3.1.1. Participants

Our multidisciplinary expert advisory panel is detailed in Table 1.

#### 3.1.2. Designing Strategies for Adoption, Implementation, and Sustainment

After reviewing the results of a prior needs assessment [13] (task 1), the expert advisory panel identified and reached a consensus on four key barriers. Barriers included: (1) low awareness of the evidence behind OAPs, (2) difficulty in measuring and addressing nonadherence, (3) complexity of a structured adherence intervention, and (4) competing priorities, changing responsibilities, and cost (Table 2).

In task 2 of the IM, the expert advisory panel identified program outcomes and objectives for adopters (pharmacy leaders, cancer center operations director, and physician leaders), for implementers (clinical pharmacist, nurse, prescriber, specialty pharmacy team, medication access technician, and social worker), and for the sustainer (cancer quality improvement committee). As detailed in Table 3, these outcomes and objectives defined the key personnel and teams responsible for initiating and maintaining key aspects of the adherence program and respective actions needed for successful implementation.

In task 3, we used a multipronged approach to select a broad list of implementation strategies. First, we reviewed each objective from task 2 and defined change objectives focusing on the following constructs: knowledge, skills, self-efficacy, outcome expectations, and other barriers from task 1. Next, the study team compiled a list of theoretical and evidence-based methods known to target the specific constructs from the literature. We relied on IM behavior-change method taxonomy (Kok et al.) to select a specific method (i.e., strategy), as well as a practical application (i.e., how the strategy is operationalized) and relevant parameters (i.e., how/when the strategy can succeed) [16]. Through this approach, we identified seven strategies for adopters, nine strategies for implementers, and four strategies for sustainers (Table 4). The expert advisory panel voted to carry out six total strategies (two each for adoption, implementation, and sustainment) for production.

In task 4, the study team collaborated with the expert advisory panel to design the documents and materials required to create a finalized implementation toolkit (Table 5). The finalized strategies included: (1) a memorandum of understanding (MOU) document to be signed by leaders and marketing materials (adoption); (2) a data-driven presentation on benefits of the program (adoption); (3) a list of standard operating procedures (implementation); (4) a motivational interviewing course (implementation); (5) electronic documentation templates with discrete fields (sustainment); and (6) an outline of key performance indicators (sustainment). For task 5, we proceeded with evaluating key implementation outcomes in phase 2 of the study (feasibility, acceptability, and appropriateness).

### 3.2. Phase 2: Feasibility, Acceptability, and Appropriateness Assessments

A total of 34 people responded to the survey (RR = 33%). Among those, 10 completed a semi-structured interview. Table 6 describes the survey and interview participants.

The six strategies identified during the IM process (phase 1) are used to categorize results from the mixed-methods phase. Table 7 provides the average (standard deviation) rating for each subdomain of the survey, along with illustrative quotes from interviews. Summaries of the findings on feasibility, acceptability, and appropriateness of the six strategies (co-developed by the expert advisory panel) are provided below.

#### 3.2.1. Strategies to Enhance Adoption

1. Memorandum of understanding between departments followed by marketing (adoption): overall, clinician survey respondents found the memorandum of understanding and other formal agreement materials to be somewhat feasible (possible: 3.9, implementable: 3.9, doable: 3.8, and easy to use: 3.6), somewhat acceptable (good match: 3.7, fitting and suitable: 3.8, and applicable: 3.9), and somewhat appropriate (appealing, likeable, and welcome: 3.8). One interview participant clarified the lack of enthusiasm for this strategy, noting, “It’s not a showstopper…it’s just important” (P7). Participants acknowledged that the purpose of the memorandum of understanding and marketing materials was to legitimize the program; one said, “The more aware that people are, the more likely they are to keep it in mind” (P4). Clinicians agreed that this strategy was important for “leadership buy-in” (P3) and to convey “a commitment to support this program” (P5).
2. Data-driven presentation (adoption): survey respondents expressed moderate enthusiasm for the data-driven presentation that would be used to persuade leaders to commit to the program across the three domains: feasibility (possible: 4.2, implementable and doable: 4, and easy to use: 3.7), acceptability (good match: 4.1, fitting and suitable: 4.3, and applicable: 4.4), and appropriateness (appealing: 4.3, and likeable and welcome: 4.4). Interview participants noted several benefits after having time to review the presentation slides, with one participant noting, “I couldn’t have said a lot of this better myself. It’s really nicely put together and effective… it absolutely lays out the importance of a model like this, it points to those hard outcomes that we can see improvement in with a model like this” (P6). Other descriptors of this strategy included: “necessary” (P1), “powerful” (P8), and “helpful” (P2).

#### 3.2.2. Strategies to Enhance Implementation

1. Standard operating procedures (implementation): Overall, survey respondents found the standard operating procedures (SOPs) to be highly feasible, acceptable, and appropriate: it ranked as the second highest of all the strategies in composite score. Individual subdomain scores included: 4.3 (possible and doable), 4.4 (implementable), and 4.2 (easy to use) for feasibility; 4.5 (fitting, suitable, and applicable) and 4.4 (good match) for acceptability; and 4.5 (welcome) and 4.6 (appealing and likable) for appropriateness. During the IDI, participants expanded on the favorable aspects of the SOPs. Notably, participants commented on the SOP being visually appealing (“I like…that this is color coded” P8), it clarifies roles and responsibilities (“the [use of] swim [lanes] is…a nice visual” P10, so as to not “have something where people are so overlapping” P9), and it effectively highlights the complexity and interconnectedness of the workflow (“highlight[ing] too how incredibly complicated medication adherence is…will make this a more successful program” P8). However, participants also cautioned the need to consider how the SOP would be operationalized and sustained. First, several clinicians emphasized the need for incremental implementation and constant iteration of the SOPs given the dynamic nature of clinical practice and the “overwhelming” (P9) nature of implementing all at once. For example, “maybe following up…a month into initiating a program…and then doing it again at three months or six months, and adapting…” P6. Second, participants noted the need to identify a program champion to ensure the SOP is implemented with high fidelity by “creating a culture” P1 that would support the workflow. Third, one participant noted the need to form “a dedicated communication channel” (P8) to facilitate a collaboration between diverse teams and avoid unnecessary duplication of efforts.
2. Motivational interview (MI) course (implementation): Compared to other strategies, there was lower level of enthusiasm for the MI course on the survey, although all scores were ≥3.25 (moderate likeability): 3.3–3.6 (feasibility), 3.7–4 (acceptability), and 3.6–3.7 (appropriateness). During the IDI, participants had a chance to review the MI course training materials (e.g., PowerPoint slides, cases, etc.). IDI participants noted positive aspects of the MI course, with one participant noting, “Yeah, from what I can see, this looks like a very useful workshop” (P6), and another noting that the approach (presentation followed by hands-on practice) “is a really good way to present it” P9. Additionally, participants stated that the course could “improve outcomes”, as it “definitely puts the patient at the center” (P6) and enables clinicians “to be more intentional about what they’re asking [by] giving them more structure” (P2). One participant noted that this course is appropriate for “physician[s]…advanced practice provider[s], and pharmacist[s]…anyone counseling a patient could absolutely benefit from this skill” (P6). Moreover, there may be an opportunity to “grow the scope…[and offer the course to] technicians… [and] CMAs (certified medical assistants)” P10. While IDI participants found the workshop feasible and appropriate, some highlighted a limited staff bandwidth, “resistant attitudes” (P8) among seasoned or experienced providers, and potential communication barriers (e.g., conducting the MI with an interpreter or during telehealth) as potential challenges of applying the MI course as a routine strategy.

#### 3.2.3. Strategies to Enhance Sustainment

1. Electronic documentation templates with discrete fields (sustainment): survey participants deemed an EHR-integrated electronic documentation template to be the most feasible (4.35), acceptable (4.59), and appropriate (4.61) strategy compared with all other strategies. Participants particularly appreciated the reduction in documentation and the ability to generate discrete data to track outcomes for key performance indicators (KPIs). The interview data support these findings. Participants liked that the proposed form had discrete fields that were “clickable” and “pre-populated”, which can “save some time” (P5). However, one participant noted that there may be some “push-back” from clinicians to require such a “constricted” documentation platform. According to one participant, “Practitioners who do things a certain way and they believe in their way are going to be less interested in following” this, although “they would probably find that over time, it’s good” P7. Participants also noted the need to consider implementation details, such as determining “where to deliver this…logistically” (P5) and visualizing the patient data on “a pretty dashboard” (P7) for all healthcare team members. The survey data revealed that participants favored the inclusion of a patient-centered financial toxicity item, scoring it notably high at 4.47. One interview participant explained, “I 100% agree” (P7) with the need to proactively ask about cost-related medication access barriers.
2. KPIs (sustainment): survey participants found KPIs to be moderately feasible (3.9–4), acceptable (4.3), and appropriate (4.3–4.5). Upon reviewing the KPIs in more detail, one interview participant noted, “I think they’re very thoughtful and tailored to a variety of…places in the process where patients might have problems, or where we as a health system could intervene” (P5). This same participant noted that using the KPIs is “feasible”, but the metrics themselves may not necessarily be “achievable” immediately: “…probably not at the beginning, but 3, 6, 12 months into the program these may be achievable measures”. The value of tracking KPIs could “help to have a pulse on how things are going in clinic and help to improve the process if necessary” and if the outcomes are improving the clinical team “could advocate for more resource[s]” P3. Another participant noted that the selected KPIs were “very strong and [would] allow [clinicians] to focus”, and went on to explain that, by “get[ting] feedback over time…maybe you reassess at six months or a year and” decide to add or remove various items (P7). Participants highlighted several areas for improvement, including rewording item on satisfaction measure (P3), analyzing data by cancer-type (P7), better highlighting how improved adherence can increase revenue to dispensing pharmacy (P7), hiring a pharmacist coordinator/technician (P5), and practicing incremental implementation (P3, P7).

## 4. Discussion

In this two-phase study, we designed and evaluated concrete strategies to enhance the adoption, implementation, and sustainability of an OAP. Using a modified IM approach, we worked closely with an expert advisory panel to produce six concrete strategies that mapped onto the six barriers we identified in our prior needs assessment. [13] In the survey, we found the greatest enthusiasm for the EHR assessment tools and SOPs, moderate enthusiasm for KPIs and data-driven presentation for leaders, and lowest enthusiasm for formal agreement materials and workshop on motivational interviewing. However, in-depth interviews that included a thorough review of the materials and documents revealed general support for all strategies, along with critical feedback on specific components.

OAP care delivery is rarely standardized. EHR assessment tools (i.e., electronic documentation templates) and SOPs that outline roles and responsibilities have the potential to standardize OAPs. In our formative needs assessment, we found that sustaining our pilot program was challenging because tasks often overlapped, creating confusion and a lack of standardized patient assessment and documentation within the EHR. As such, program implementers struggled with tracking successes and challenges. Additionally, complete and accurate documentation within the EHR could enhance patient safety [17] by facilitating communication between members of the healthcare team. Practice standards from the Hematology/Oncology Pharmacy Association (HOPA) [9] highlight the importance of building an OAP with standardized policies and procedures and with clarified roles and responsibilities.

Our team previously published the KPIs developed during the first phase of this project [18]. In collaboration with the expert advisory panel, we identified 11 KPIs, corresponding metrics, and predetermined targets when appropriate. The KPIs were organized across three domains: clinical indicators (time to treatment, adherence rate, adverse events, financial toxicity, patient satisfaction, treatment-related ED visits, and hospitalizations); operational indicators (completed patient assessment, referral to support services, and time spent in various phases of the OAP as indicated by the SOP); and economic (pharmacist billing for OAP services). In the survey and IDI, participants saw the value of having KPIs, although several suggestions were offered to improve the usability of the measures. With refinement, these KPIs could supplement the existing cancer-related quality measures (especially ASCO’s Quality Oncology Practice Initiative standards) by offering health systems OAA-specific indicators [19].

Overall, the two strategies that were focused on adoption—data-driven presentation and memorandum of understanding and marketing materials—received lower than expected enthusiasm on the survey. Notably, the survey respondents did not have the opportunity to closely review the strategies; instead, they read descriptions and were asked questions regarding their perceptions of feasibility, acceptability, and appropriateness. During the IDI, participants had additional time to review the strategies and offer detailed feedback. The data-driven presentation was prepared with feedback from expert advisory panel, and it leveraged what is already known about priority setting and resource allocation in health system settings [20]. In a 2021 scoping review, Seixas et. al. characterized the key findings that influence health system leaders’ decisions in paying for new clinical initiatives, including (1) a review of the published literature for clinical rationale; (2) data on disease prevalence/trends; (3) economic evaluations/value proposition; and (4) advocacy/involvement of clinical experts. Based on these principles, this presentation has the following sections: (1) the growing use of OAAs and need for adherence; (2) the impact of nonadherence on clinical and economic outcomes; (3) a review of the institutional adherence data and gaps in patient care/patient safety; (4) the benefit of OAPs to patients and the health system; (5) a review of available pilot data; and (6) alignment with national standards/guidelines and emerging value-based payment models. Participants had favorable impressions of the presentation as well as the memorandum of understanding and marketing materials that would enhance the likelihood of the program’s formal adoption by leaders. As IDI participants noted, formal agreements between departments—such as memoranda of understanding—can clarify roles and expectations [21], foster cooperation and reciprocity [22], and emphasize ethical accountability [23]. These concepts, rooted in social exchange theory, enable organizations to successfully adopt and even sustain interventions [24,25,26,27,28,29].

The motivational interview course received the lowest enthusiasm with regards to feasibility, acceptability, and appropriateness. In both the survey and the IDI, participants highlighted the theoretical benefits of having an MI course to enhance clinicians’ capacity to ask patients about adherence and to navigate a conversation regarding barriers. However, participants expressed that finding time to complete the MI training would be difficult given the stressful workload that many oncology professionals face [30,31,32,33]. In a discussion with the expert advisory panel, a possible solution for this could be providing MI training during already existing team meetings (e.g., weekly huddles, division/departmental meetings, etc.). This approach has been shown to increase the successful completion of required training by staff without taking additional time [34,35,36,37,38].

There were two major limitations to this study. First, our study was conducted within one integrated health system in North Carolina. Although we worked to integrate a diverse set of clinicians and administrators across academic and community cancer centers, an adaptation of these strategies may be needed to enhance the generalizability. Second, we did not engage patients or caregivers in this study. Initially, we did identify two patients who agreed to join our expert advisory panel; however, our study team felt that their engagement would be more valuable at the next phase of this study (i.e., adherence program refinement).

## 5. Conclusions

Overall, our expert advisory panel-driven strategy design approach with the IM was positively received by oncology professionals. In the next phase of our study, the six strategies identified above will be packaged into a cohesive intervention bundle and tested prospectively in a future hybrid effectiveness implementation trial.

## Figures and Tables

**Table 1:** Expert advisory panel participants.

Setting	Role	Number
Academic Medical Center	Hematologist/Oncologist	2
	Nurse Practitioner	1
	Clinical Pharmacist	2
	Hospital Administrators **	5 **
	Social Worker	1
	Information Technology Professionals	3
Community Cancer Center	Hematologist/Oncologist	1
	Clinical Pharmacist	2
	Pharmacy Manager	1
Specialty Pharmacy *	Pharmcy Managers	1

* Specialty pharmacy serves both academic and community cancer centers. ** Cancer center director, nursing manager, pharmacy department director, pharmacy clinical manager, and medication assistance program coordinator.

**Table 2:** Importance and changeability of barriers according to members of the expert advisory panel (n = 12).

Barriers *	Importance ** (Mean ± SD)	Category ***	Changeability ^+^ (Mean ± SD)	Category ***
Low awareness of the evidence behind OAPs	3.67 ± 0.94	Moderately–very important	3.25 ± 1.09	Neither easy nor difficult–somewhat easy
	Top 2 Box Score: 8/12 = 67%	Important	Top 2 Box Score: 7/12 = 58%	Easy
Difficulty of measuring and addressing nonadherence	4.42 ± 0.76	Very–extremely important	2.17 ± 0.69	Somewhat difficult–neither easy nor difficult
	Top 2 Box Score: 10/12 = 83%	Important	Top 2 Box Score: 1/12 = 8%	Difficult
Complexity of a structured adherence intervention	4.00 ± 0.82	Very important	2.33 ± 0.62	Somewhat difficult–neither easy nor difficult
	Top 2 Box Score: 10/12 = 83%	Important	Top 2 Box Score: 0/12 = 0%	Difficult
Competing priorities, changing responsibilities, and cost	4.33 ± 0.94	Very–extremely important	1.58 ± 0.95	Extremely–somewhat difficult
	Top 2 Box Score: 10/12 = 83%	Important	Top 2 Box Score: 1/12 = 8%	Difficult

* Barriers from our prior qualitative interviews [13]. ** Importance is defined as the degree to which a particular barrier is considered to be a priority for action according to the expert advisory panel using a Likert-type scale (1 = Not Important; 5 = Extremely Important). ^+^ Changeability is defined as how easily that a particular barrier is likely to be modified or removed according to the expert advisory panel using a Likert-type scale (1 = Extremely Difficult; 5 = Extremely Easy) *** Dichotomized categories: mean and SD are provided for all Likert-type questions. If more than 50% of respondents selected either 4 or 5 on the instrument scale (i.e., “Top 2 Box Score”), the barrier was classified as important or easy, respectively. OAP = oral anticancer agent adherence support program.

**Table 3:** Outcomes and performance objectives and agreement results from expert advisory panel.

Adoption Phase			
Target: Role	Outcomes	Performance Objectives	EAP Agreement * (Mean ± SD)
**Pharmacy leadership:** *adopter*	Managers decide to adopt the OAA adherence program formally in collaboration with cancer center and physician leaders	Agree to participate in adherence program Agree to participate in evaluation Designate implementer and champion	5 ± 0
**Cancer Center operations director:** *adopter*	Director decides to adopt the OAA adherence program in collaboration with physician and pharmacy leaders	Agree to participate in adherence program Designate champion Gain support from key partners	4.26 ± 1.02
**Physician leadership:** *adopter*	Physician leader decides to adopt the OAP adherence program in collaboration with pharmacy and physician leaders	Agree to participate in adherence program Agree to participate in evaluation Designate champion	4.54 ± 0.73
**Implementation Phase**			
**Target: role**	**Outcomes**	**Performance Objectives**	EAP Agreement * **(mean ± SD)**
**Clinical pharmacist:** *implementer*	Pharmacist will fully implement OAA adherence program	Conduct and document adverse event and adherence assessments Counsel and document patients on adverse event(s) and adherence methods Identify high-, medium-, low-risk adherence patients Monitor and follow patients clinically, regardless of pharmacy status (internal and external pharmacy)	4.64 ± 0.5
**Nursing team:** *implementer*	Nurse will co-implement OAA adherence program with pharmacist	Triage phone calls and electronic messages from patients in-between scheduled patient assessments Determine need to escalate care to other members of healthcare team Training on supporting patients with adherence and symptom management Document care with patients and notify pharmacist	4.57 ± 0.73
**Physician/APP:** *implementer*	Physician will refer patients to OAA adherence program	Ensure that patients on oral chemotherapy are enrolled in adherence program	4.71 ± 0.45
**Specialty pharmacy team:** *implementer*	Specialty pharmacy team will complete refill/adherence calls	Conduct telephone refill/adherence calls Training on cancer subtypes and relevant drugs Pharmacy technicians escalate nonadherence calls to pharmacist	5 ± 0
**Medication access technician:** *implementer*	Technician will address financial barriers to oral chemotherapy	Accept referrals from physicians, nurses, pharmacists, and social workers Triage referrals based on priority (urgency) and risk of nonadherence Apply and annually renew prior authorizations and manufacturer assistance applications	4.71 ± 0.7
**Social worker:** *implementer*	Social worker will identify financial and psychological barriers	Refer medication access related concerns to medication access technician Broadly assess and address financial barriers and associated psychological stress	4.53 ± 0
**Sustainment Phase**			
**Target: role**	**Outcomes**	**Performance Objectives**	EAP Agreement * **(mean ± SD)**
**Cancer center QI committee:** *Sustainer*	Ensure clinic leadership maintains the OAA adherence program as part of standard practice	Assemble standing meeting with key stakeholders, champions, and leadership Assure adherence rates and nonadherence reasons continue to be reported (and remain stable or on upward trend)	5 ± 0

* EAP Agreement is defined as the degree to which each individual EAP participant agrees with the consensus definitions of roles, outcomes, and performance objectives measured on a Likert-type scale (1 = Strongly Disagree; 5 = Strongly Agree). EAP = expert advisory panel; OAA = oral anticancer agent.

**Table 4:** Distinct strategies proposed by the study team and then selected by the expert advisory panel via consensus-building discussions.

Strategies for Adoption			
Roles	Outcome Summary	Strategies Proposed (IM Step 3)	Strategies Selected for Production (IM Step 4)
Pharmacy, physician, and cancer center leaders	Leaders will formally agree to adopt an OAP	Evaluate readiness using the Organizational Readiness for Change (ORIC) tool Data-driven presentation on the benefits of an adherence program to leaders Public announcement (e.g., newsletter) and possible signed agreements among relevant stakeholders Setting a go-live date Outline key performance indicators 1:1 meetings with potential implementers and champions Integrate support from IT	Data-driven presentation of the benefits of an adherence program to leaders Public announcement (e.g., newsletter) and possible signed agreements among relevant stakeholders Outline key performance indicators
**Strategies for Implementation**			
**Roles**	**Outcome Summary**	**Strategies Proposed (IM Step 3)**	**Strategies Selected for Production (IM Step 4)**
Physician, APP	Providers will refer patients to the oral chemotherapy program	Weekly team huddles to debrief on new start oral chemotherapy Leverage IT team to passively (not pop-up) remind providers about referral to program when prescribing oral chemotherapy Initial education about program workflow (~45 min) with quarterly multidisciplinary reminders at already existing team meetings (~15 min)	N/A
Pharmacy and nursing teams	Pharmacists and nurses will assess and address adherence, manage adverse effects, and document encounters Specialty pharmacy team will complete refill and adherence calls	Short course on the impact of adherence on clinical outcomes, barriers to adherence (e.g., socioeconomic and illiteracy), and motivational interviewing techniques Standardized adherence assessment integrated into EHR (using “dot phrase” or as Epic “smart form”) Develop process map and standard operation procedure (including internal vs. external specialty pharmacy, risk stratification, and escalating care) Quarterly team huddle	Short course on the impact of adherence on clinical outcomes, barriers to adherence (e.g., socioeconomic and illiteracy), and motivational interviewing techniques Standardized adherence assessment integrated into EHR (using “dot phrase” or as Epic “smart form”) Develop process map and standard operation procedure (including internal vs. external specialty pharmacy, risk stratification, and escalating care)
Medication access technician and social work	Medication access technician will accept referrals and address financial barriers to OAAs Social work will support technician and address psychological stress associated with socioeconomic barriers	Initial education about program workflow (~45 min) with quarterly multidisciplinary reminders at already existing team meetings (~15 min) Develop process map and standard operation procedure (including internal vs. external specialty pharmacy, risk stratification, and escalating care)	Develop process map and standard operation procedure (including internal vs. external specialty pharmacy, risk stratification, and escalating care)
**Strategies for Sustainment**			
**Roles**	**Outcome Summary**	**Strategies Proposed (IM Step 3)**	**Strategies Selected for Production (IM Step 4)**
Cancer center QI committee	Ensure oral chemotherapy program is maintained as part of standard practice	Develop process map and standard operation procedure Educational meeting on program goals and objectives Define measurable performance indicators and metrics Leverage IT/data analytics teams to set up dashboards for reporting relevant KPIs and metrics	Develop process map and standard operation procedure Define measurable performance indicators and metrics

IM = implementation mapping; OAP = oral anticancer agent adherence support program; IT = information technology; EHR = electronic health record; OAA = oral anticancer agent; QI = quality improvement; KPI = key performance indicator.

**Table 5:** Strategies matched to identified barriers.

	Barrier	Strategy
Adoption	Low awareness of the evidence behind OAPs	Signed MOU between departments (pharmacy and cancer center) and marketing
		Data-driven presentation on benefit of OAP to patients and health system
Implementation	Complexity of a structured adherence intervention	Standard operating procedure
	Difficulty of measuring and addressing nonadherence	Motivational interviewing course
Sustainment		Design EHR documentation templates (i.e., “smart” forms)
	Competing priorities, changing responsibilities, and cost	Key performance indicators defined

OAP = oral anticancer agent adherence support program; MOU = memorandum of understanding; EHR = electronic health record.

**Table 6:** Phase 2 (mixed-methods) participants.

Survey Participants		
Characteristic		*N* (%)
Role		
Physician		17 (51.5%)
Pharmacist		8 (24.2%)
Advanced Practice Provider		6 (18.2%)
Nurse		1 (3%)
Social Worker		1 (3%)
Experience		
1–5 years		13 (40.6%)
6–10 years		7 (21.9%)
>10 years		12 (37.5%)
Setting		
Academic Medical Center		23 (71.9%)
Urban Community Cancer Center		7 (21.9%)
Rural Cancer Center		2 (6.3%)
Total		33
**Interview Participants**		
Setting	Role	N
Academic	Hospital Administrator	2
	Pharmacist	1
	Hematologist/Oncologist	1
	Nurse Practitioner	1
Urban (community)	Pharmacist	3
	Director	1
Rural (community)	Pharmacist	1
Total		10

**Table 7:** Joint display of quantitative and qualitative findings.

	Quantitative Results (Composite Score *)			Qualitative Results (Illustrative Quotes)	Meta Inference (Alignment)
Adoption Strategies					
	Feas.	Acc.	Appr.		
Fromal agreement materials *	3.78	3.76	3.76	“It’s not a showstopper… it’s just important” P7 “I don’t think it’s a bad idea. I think the more aware that people are, the more likely they are to keep it in mind. Anytime you send out something formal, it opens it up to be, ‘Hey, this is legit and we need to refer patients to this program’”. P4	(Enhance) Agreement materials (e.g., MOU and marketing) are key to receiving a formal adoption and endorsement of an OAP
Data-driven presentation	3.98	4.26	4.33	“I think the level of how it’s being presented, I think is appropriate to most individuals that would need to see this. I don’t think any major changes on that would need to be made”. P8	(Confirm) A data-driven presentation on the benefits of an OAP program to patients and the institution (e.g., practice, health system, and pharmacy) can be effective if presented to key decision makers
Implementation Strategies					
Standard operating procedure	4.29	4.49	4.58	“They’re very thoughtful, they’re very detailed—I think the swim lanes as you have them designating the different team members and their responsibilities is the most [helpful] because I think that aids to visually see the roles and responsibilities to which I think the [clinical pharmacist] would be interested in because a lot of us would just kind of own it all, you know? So, the visual distinction of the role the nurse navigator can play, the physician, etc., in these swim lanes, and color-coded—I think that’s a nice visual to recognize that, or to identify that”. P10	(Confirm) Standard operating procedures can be effective in clearly outlining roles and esponsibilities for an otherwise complex OAP workflow
Motivational interview course	3.38	3.88	3.65	“Motivational interviewing’s a skill. I grew up…playing basketball and if I just told you for 30 min this is how you shoot a free throw, it wouldn’t help you shoot a free throw any better. You have to physically do it and do it routinely to get better at it. So, at the very least seeing someone shoot a free throw would be more helpful than reading or hearing people talk about shooting free throws. Same thing for motivational interviewing and we do this with our students in the curriculum. We have standardized patients, and we have them practice motivational interviewing from the very first year”. P1	(Confirm) Although motivational interviewing skills are important for an adherence program, a one-time workshop alone may not be effective in enhancing this skill among health care professionals
Sustainment Strategies					
EHR documentation templates with discrete fields	4.35	4.59	4.61	“Like with the assessment forms that you’ve created, they’re all measurable, and they should all give insight into the patient population you’re following. And then I think also like you may have mentioned will just help to have a pulse on how things are going in clinic and help to improve the process if necessary”. P3	(Confirm) EHR documentation templates with discrete fields can generate the data needed for key performance indicators while saving clinicians time on documentation
Key performance indicators	3.99	4.31	4.39	“I think having this is very strong and allows you to focus…I think you have a good mix of things that impact patients, trying to do some things that impact overall cost of care and adherence, and you know, you can’t have everything, or else you’re going to have 12 pages of forms… maybe you reassess at six months or a year and find out, “We should add this, we should take this away”. But it looks like a really great start to me”. P7	(Confirm) Focused KPIs that track clinical and economic outcomes can be useful in demonstrating program effectiveness. Addressing identified gaps can also facilitate sustainability

* Composite (average) score for feasibility (Feas.) components (possible, implementable, and doable); acceptability (Acc.) components (good match, fitting, and suitable); and appropriateness (Appr.) components (appealing, likeable, and welcome). MOU = memorandum of understanding.

## Data Availability

The data presented in this study are available on request from the corresponding authors due to participant privacy.
